# 2431: Characterization of immune cell differences with anti-thymocyte globulin (ATG) and granulocyte colony stimulating factor (GCSF) in both preclinical and clinical models of type 1 diabetes

**DOI:** 10.1017/cts.2017.136

**Published:** 2018-05-10

**Authors:** Andrea Lin, Clayton Mathews, Michael Haller, Todd Brusko, Mark Atkinson, Ryan Flynn

## Abstract

OBJECTIVES/SPECIFIC AIMS: Understand the immunomodulatory effects of anti-thymocyte globulin (ATG) and granulocyte colony stimulating factor (G-CSF) on type 1 diabetes patients using samples and in the preclinical model, the nonobese diabetic mouse. METHODS/STUDY POPULATION: Flow cytometry analysis of phase 1 peripheral blood samples treatment of nonobese diabetic mouse with ATG and GCSF and flow cytometry analysis of immune organs (spleen, lymph nodes, blood, bone marrow). RESULTS/ANTICIPATED RESULTS: Changes in both innate and adaptive immune cell subsets including plasmacytoid dendritic cells, naive, memory, effector CD4+ and CD8+ T-cells, and CD4+ T-regulatory cells and CD8+ T-regulatory cells DISCUSSION/SIGNIFICANCE OF IMPACT: Understanding of immune cell targets for immunotherapy in new-onset type 1 diabetes patients.

